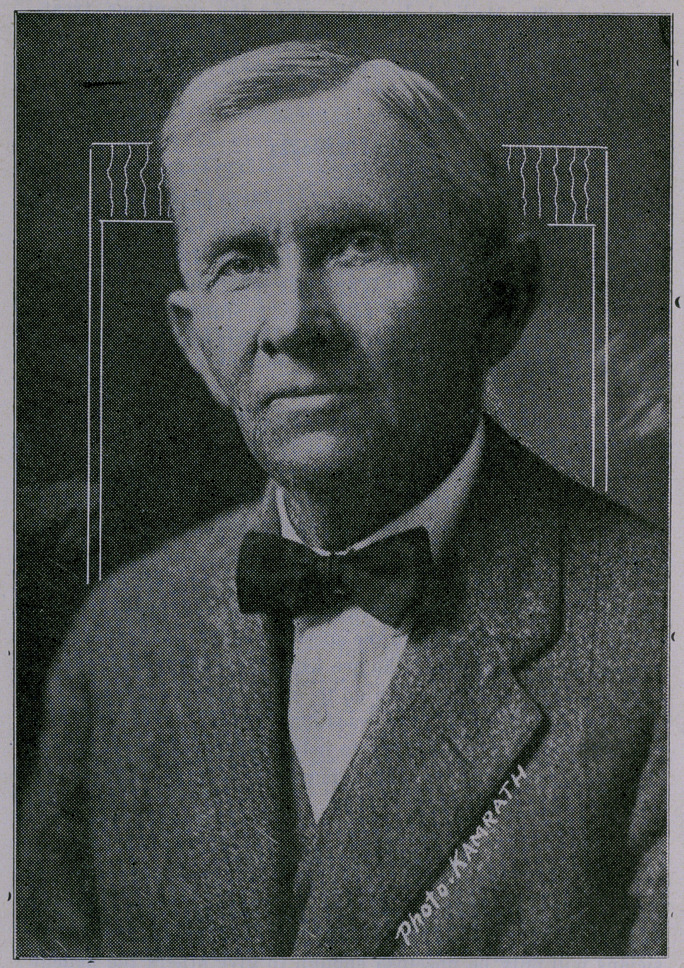# Dr. W. D. Yett, Mayor

**Published:** 1919-04

**Authors:** 


**Dr. W. D. Yett, Mayor.**

---

Dr. W. D. Yett, retired physician, capitalist and banker, has been elected to succeed Hon. A. P. Wooldridge as mayor of Austin. This is a distinct honor conferred on Dr. Yett, as he has been called to succeed one of the best mayors of the South, a man who has been responsible for making Austin one of the most beautiful cities of the country. The medical profession of the State will watch with much interest the career of Mayor Yett and will take pride in his achievements as mayor of Austin.

Dr. Yett is a Texas product, having been born and reared in Burnet County, near Marble Falls. After completing a high school education he graduated from the old Texas Medical College, winning the faculty gold medal. Later he graduated from the Medical Department of Vanderbilt University. He then practiced medicine a number of years in Marble Falls. He was elected to represent his senatorial district in the legislature and served in the Twenty-fifth, Twenty-sixth and Twenty-seventh Legislatures. When the Citizens State Bank of Marble Falls was organized twelve years ago, he was elected president of that institution, which position he still holds. He moved to Austin six years ago to take advantage of the opportunities afforded by the University of Texas for the education of his children, and has since then been interested deeply in all that pertained to the development of the city.

## Figures and Tables

**Figure f1:**